# Role of multidimensional factors in the diagnosis and treatment of tonsillopharyngitis in primary care: a qualitative study

**DOI:** 10.1186/s12875-022-01881-x

**Published:** 2022-11-04

**Authors:** Ieva Rutkovska, Zane Linde-Ozola, Elita Poplavska

**Affiliations:** 1 Department of Applied Pharmacy, Faculty of Pharmacy, Riga Stradiņš University, Riga, Latvia; 2 Department of Anthropology, Faculty of Humanities, University of Latvia, Riga, Latvia; 3 Institute of Public Health, Riga Stradiņš University, Riga, Latvia

**Keywords:** Tonsillopharyngitis, General Practitioners, Qualitative Study, Decision-Making Process

## Abstract

**Background:**

Tonsillopharyngitis is one of the most frequently observed upper respiratory tract infections, for which antibiotics are prescribed in ambulatory care. In most cases, tonsillopharyngitis is benign and self-limiting, mostly a viral condition. The aim of this study was to explore the diagnostic and treatment process of tonsillopharyngitis by general practitioners and to understand decisions regarding antibiotic prescribing and the factors that shape these practices.

**Methods/design:**

This was a qualitative interview study in primary care practices in Latvia. Semi-structured face-to-face interviews were conducted with general practitioners from November 2016 to January 2017. Thematic analysis was applied to identify factors that influence the prescribing practice in a primary care setting in conjunction with a specific context in which the prescriber practices.

**Results:**

Decisions and practice of general practitioner are not static over time or context; they occur within an environmental setting influenced by individual factors of general practitioners, the health care system, and practice-specific factors that shape the diagnosis and antibiotic prescribing in the tonsillopharyngitis. Interviewed general practitioners rely primarily on their personal experience, perception, and skills acquired in their practice, which are encouraged by the environment, where the necessary tools and resources are not in place to encourage rational prescribing of antibiotics.

**Conclusions:**

General practitioners’ decision regarding antibiotic prescribing is an unstable concept that differs between prescribers. The health care system could augment the experience of general practitioners through structural changes such as guidelines, availability of antibiotics, and available antibiotics package size.

## Background

Tonsillopharyngitis (TF) is a frequently observed condition with symptoms of pharynx inflammation. In most cases, TF is a benign, self-limiting, and viral condition (40–80%). Bacterial causes include group A beta-haemolytic streptococci (GABHS) (15–30%) and group C beta-haemolytic streptococci (5%) [1–3]. Symptoms of bacterial and viral TF can last around one week, but most patients improve without antibiotics (ABs), regardless of the cause [4].

Only in cases of GABHS infections AB treatment is recommended [5]. For the standard GABHS TF, 10 days of treatment with phenoxymethylpenicillin is recommended [1, 6] to reduce possible nonsuppurative complications – acute rheumatic fever [7, 8]. A shorter course may be sufficient if ABs are indicated for symptomatic, not microbiological, cure. Although in Latvia relatively low AB prescribing is observed in the outpatient setting [9], TF is one of the most frequently treated conditions with ABs [10].

In developed countries, complications of TF have become rare [11–13] and the rates of complications do not differ with or without AB therapy [5, 12, 14, 15]. The difficulty in deciding regarding the AB use arises from the fact that ﻿it is hard to differentiate between the ‘bad’, ‘good’ and ‘harmless’ versions of GABHS as pointed out by Gröndal (2018) [16].

There are different tools for the diagnosis of TF and the choice of the correct treatment method. For TF diagnostics, several point-of-care tests and clinical scoring systems are available, such as rapid strep tests, Centor score, McIsaac score and FeverPAIN score [4, 17, 18]. However, scoring tools do not allow to rule out or affirm the GABHS TF [19, 20].

The disadvantage of rapid strep tests is that they do not exclude patients who may be carriers who do not require treatment. [21] For this reason, rapid tests are recommended in conjunction with clinical scoring systems [22].

Important tool for general practitioners (GPs) in the diagnoses and treatment of TF is clinical guidelines. ﻿Most European countries have national guidelines on AB treatment of TF for children and adults, but recommendations differ greatly ﻿ [23]. In Latvia, in the case of TF, official recommendations on rational AB pharmacotherapy are only available for children [24]. Largest hospitals have released their own recommendations for empirical antimicrobial treatment, but for some indications, including TF treatment recommendations differ [25, 26]. However, national guidelines on AB treatment still are not available in Latvia.

Qualitative studies suggest that physicians use different strategies to manage TF and uncertainty related to AB prescribing. For example, a study that evaluated the use of rapid streptococcal antigen detection tests [27] found that GPs used the test to confirm a diagnosis and support their prescribing decision, but at the same time, they questioned the utility of this test. A study in Sweden concluded that GPs excessively use guidelines and point of care tests to reduce uncertainty. However, most of the GPs believed that they did not have difficulties in managing TF [28].

Previous qualitative studies on this topic have focused on individual factors (eg, knowledge, attitude) related to diagnosis or AB prescribing in TF separately; however, this could not show the complexity and interplay of multiple factors in the decision-making process of the GP for diagnosis and treatment in different contexts. In particular, research from low-middle-income countries has made visible how structural factors (eg, economic concerns, availability of ABs) shape ‘individual’ decision-making practices of ABs. Research on exploring individual and structural factors as interlinked in the use and overuse of ABs is an important knowledge gap [29, 30].

The aim of this study was to explore the diagnostic and the treatment process of GPs of TF and factors that shape these practices in Latvia. We intend to contribute to an emerging scholarship that brings together individual and structural factors as interlinked when critically unpacking AB use and misuse practices.

## Methods

A qualitative study was conducted using semi-structured interviews with GPs. For our research question, individual interviews were chosen instead of focus groups because:

- 1) for practical reasons, as it is easier to adjust interview time and place for research participants who are very busy, thus encouraging greater participation in research.
- 2) the interviews provided a more relaxed and anonymous setting, providing an environment for research participants to speak more freely and without worrying about other professionals' opinions or judgements, especially if these differ.

A semi-structured interview guide was developed with questions exploring the practices and decisions of GPs regarding the TF diagnostic process and AB prescribing.

### Study design and participant recruitment

GPs were recruited using purposive sampling. A GP was eligible for this study if he or she practised in the capital city of Latvia, had at least 1000 patients per practice, provided services to children and adults, and consented to participate in this study.

Based on information in the public NHS electronic database of registered GPs in Riga [31] we created a list of GPs using maximum variation sampling considering the location of the practice (one to two participants from each of the six districts of the city). We contacted practitioners in the order in which they were listed in the database at least twice at different times. If the GP could not be reached or he/she declined participation, we contacted the next person in the list. In total, 36 calls were made to recruit 8 participants. Of 36 calls in half of the cases, we were unable to reach the GP after two telephone calls. Among those who declined to participate, the most common reasons were lack of time and not wanting to participate in the interviews. All GPs interviewed signed an informed consent form.

### Data collection

Semi-structured individual face-to-face interviews were conducted from November 2016 to January 2017. After developing an interview guide, it was piloted during the first two interviews. These interviews are included in the analysis, as the guide had only minor revisions. All interviews were conducted in general practice sites that lasted 25 min to an hour. Interviews were conducted by one trained interviewer (I.R.). Before the interviews were completed, the interviewer had no personal relationship with any of the interviewees. Interviews were digitally recorded and transcribed verbatim. To maintain the confidentiality of the interviewees, the interviews were pseudonymised, and we assigned an identification code to each interview.

Data collection continued until data saturation was reached. Data saturation was reached after six interviews when no new codes appeared. Additionally, two more interviews were conducted.

The characteristics of eight participating GPs are summarized in Table 1.

**Table 1:** Characteristics of the GPs interviewed

Name/ID	Gender	Years in GP practice	Patients in practice (adults and children)
GP1	Woman	> 10	> 3000
GP2	Woman	> 5	> 2500
GP3	Woman	< 5	> 1100
GP 4	Woman	> 10	> 3200
GP 5	Man	> 10	> 2100
GP 6	Man	< 5	> 1200
GP 7	Woman	> 10	> 1800
GP 8	Woman	> 10	> 1400

### Data analysis

The interview transcripts were uploaded to Atlas.ti for data analysis, which helped to manage the qualitative data. Inductive thematic analysis was performed as described by Braun and Clarke [32] with aim to determine the diagnostic process of TF by GPs, decisions about AB prescribing, and factors that shape these practices. Six phases of thematic analysis were followed, including (1) familiarizing the data, (2) generating initial codes, (3) searching for themes and assigning codes to these themes, (4) reviewing themes through the data analysis process that continued with every new transcribed interview, (5) defining and naming themes, and (6) producing the data analysis [32]. Patterns and prominent themes that appeared consistently in the data were identified and labelled with codes after each interview, and data collection was continued until data saturation was reached. Codes and their definitions were refined during the analysis process in order to produce a set of themes and subthemes, which explained the TF diagnostic process by GPs and decisions regarding AB prescribing, as well as the underlying factors shaping these processes. We did not provide an opportunity to see the results of the analysis to the study participants, as this method was not applied in this study. All authors have available all of the interview transcripts to become familiar with the data. Using Atlas.ti software, I.R. carried out the first cycle of coding where afterwards all authors discussed the emerging codes, categories and themes, and grouped the higher-order headings into sub-categories, for example in the first cycle of coding the three-step decision-making process was developed but ecology of factors influencing the decision-making process was developed during the discussions among all authors as part of secondary cycle of coding and analysis. This process was continued until all authors agreed on the final structure and content of data analysis.

## Results

**Fig. 1:**
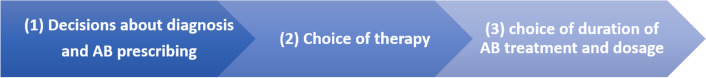
TF diagnostic and treatment process by GPs

We identified that the decision-making process regarding AB prescribing can be explained as a three-step decision-making process: (1) decision about the diagnosis and AB prescribing, where GPs identify if a patient has TF, differentiate bacterial from viral TF, and decide if a patient needs an AB treatment; (2) choice of therapy, where doctors decide which AB is indicated in patients with TF; and (3) choice of duration of AB treatment and dosage (Fig. 1).

In each of these decision-making steps, we identified individual, practice-specific, healthcare system factors that shape the diagnostic and treatment process of TF.

### Decisions on diagnosis and AB prescribing

#### Individual perception of TF management

All GPs interviewed dealt with TF on a daily basis and TF was perceived as a simple and mundane disease. Diagnosis of TF was not considered a very difficult task and interviewed GPs had general confidence in managing patients with TF:

“I do not use any diagnostic criteria; I do not calculate a score because I think it is not complicated [TF diagnosis]. During the study period at the university, it was highlighted in lectures that this diagnosis should not cause any difficulties.” (GP-5)

While reflecting on the diagnosis of TF, interviewed GPs were not concerned about possible complications of bacterial TF. In their opinion, the main objective of AB therapy was to reduce symptoms. GPs’ confidence was largely attributed to their past clinical experience. Furthermore, the interviewees believed that the diagnosis could be made based on their suspicion and inner guidance or gut feeling that was used in conjunction with the objective examination and patient complaints.

“There is a clinical picture in front of me. Perhaps the patient may not have anything after those Centor criteria because it is purely mathematical, but when I see that person, I can feel if he has a bacterial TF.” (GP-6)

Although clinical experience is a useful way of diagnosing, in cases of uncertainty it can lead to false results. To mitigate uncertainty, some GPs used rapid strep tests (RSTs). The interviews showed that the GPs who used RST were more certain of the diagnosis and therefore felt more confident in avoiding AB prescribing in cases of negative RST results.

“If there is a slight suspicion, I use the GABHS rapid antigen detection test, if the result is negative, then I calm down my patient, follow up, and do not give antibacterial therapy.” (GP-4)

GPs reported using RSTs in some cases, but when it is not used in conjunction with clinical scoring systems or microbiological tests, it can lead to false positive results for GABHS carriers. In these situations, an RST alone would not help clarify the diagnosis of TF and would only increase the uncertainty.

“There are children who have TF repeatedly[...], I tried to learn about this in seminars, I have purchased RST, now I check all patients in a row, but the more I check, the more I am confused with those streptococci. There have been many times when I think there would not be a strep throat because it looks completely like virus-induced TF, but I take RST and it is positive.” (GP-8)

The CRP express test was not used among interviewees and only one of the interviewed GPs was aware of the clinical scoring systems but did not use it. The interviewed GPs also did not use microbiological tests, because they felt that waiting for results up to 24–48 h is cumbersome and would delay their work.

#### Specific GPs practice-level factors

Two specific factors at the practice level of GPs were shaping decisions: (1) perception of occupation pressures and (2) perception of maintaining relationships with patients. In some cases, interviewed GPs preferred to prescribe ABs because they believed that prescribing ABs saves time and makes their workflow more efficient. For example, after prescribing ABs, it is not necessary to make patients come back for another visit to manage TF.

“I look at the person and try to predict how much he wants antibiotics. Well, we are tired and cannot deal with them.” (GP-3)

As a result, time pressure and occupational fatigue can lead to overprescribing of ABs when dealing with TF cases. Similarly, the perception of maintaining relations with patients also shaped decision to prescribe ABs. All interviewed GPs considered patients’ expectations. For example, if a patient wanted to use ABs in typical viral TF, the GPs usually tried to talk to the patient, but if he or she insisted, the GPs tended to prescribe ABs. Patients expected that ABs will provide quick infection management and a reduction of sick leave to a minimum.

“[…] people want to get back to work quickly, and I cannot say to them "Just walk with this, maybe it is viral". Maybe antibiotics are prescribed more often than they should be because with antibiotics patients could quickly get back to work and will have no complications.” (GP-4)

In some cases, to deal with patients who insist on the use of ABs, delayed prescribing was used. GPs try to manage these situations in a way that reduces the harm to the patient, while at the same time ensuring in his opinion the best possible outcome for the patient. From the examples mentioned by GPs, it appeared that this could result in decisions that are not in agreement with current best clinical practice. All interviewees acknowledged the contradiction they faced in their daily practice.

#### Availability of national guidelines

As mentioned above, national recommendations are available only for pediatric population. GPs did not routinely use clinical guidelines, but one of the interviewees felt the need for them in his practice. He said in the interview that if guidelines were implemented and applied in daily practice, it would help GPs to reduce uncertainty and variability in prescribed treatments. According to him, it would have a good impact on decisions regarding AB prescribing, because it would standardize the care of TF patients and reduce the possibility that patients visit several GPs with the same diagnosis in search of AB prescriptions.

“I think if there were guidelines, which everyone used, then it would be much easier for us (GPs) and for patients. The patient with one disease would go to different doctors and have the same diagnosis and treatment. It would reduce the waiting time for appointments. And then it would be easier for us to work because right now national guidelines are not available. I do not have guidance, but I have responsibility.” (GP-6)

On the contrary, other GPs felt no need for clinical guidelines because they perceived them as too long and cumbersome to apply in daily practice. GPs did not perceive the value of routinely using and consulting guidelines. Some interviewees felt that guidelines might restrict their autonomy to practice and that they do not support ‘guideline-guided medicine’.

“Guidelines would not be implemented in everyday practice, let us face it, that is not going to happen. Here we do not have guideline-guided medicine.” (GP-2)

### Choice of therapy

When choosing the specific AB for TF treatment, the decision was shaped by (1) individual GP beliefs and previous experience of effectiveness of different ABs and (2) affordability and availability of ABs within the healthcare system.

#### Beliefs and experience of the effectiveness of ABs

Opinions on the effectiveness of ABs varied greatly between GPs. Interviewed GPs relied on their personal experience to judge the effectiveness of different ABs.

Some of the interviewed GPs did not question the effectiveness of phenoxymethylpenicillin (POMP) in cases of GABHS, but many GPs thought that POMP is ineffective for the treatment of bacterial TF. From interviews it was apparent that if there were cases where a specific AB did not help in TF, then there is a very small possibility that GPs are going to use the same AB in another situation.

“POMP does not work, it acts inconclusive, and it deceives. In any situation in which I have tried it, it is much less effective and not persuasive.” (GP-7)

Interviewed GPs also noted that it would be desirable that patients do not need to change their treatment with ABs later. As a result, this rationale led mainly to prescribing broad-spectrum ABs.

Opinions on the effectiveness of amoxicillin and amoxicillin with clavulanic acid also varied: there were views that amoxicillin is ineffective and in cases of bacterial TF, amoxicillin with clavulanic acid should be used, but some believed that its effectiveness is similar, yet amoxicillin alone has fewer side effects.

“[...] It goes so well with azithromycin – on day 2 they feel fine, but if I follow the guidelines and prescribe amoxicillin, they still feel sick on day 2 and 3, but with azithromycin - ‘Doctor, I feel fine, this is a miracle cure’ (…) Every doctor uses something [AB] that is familiar and more tested in his own practice.” (GP-8)

Individual beliefs and expectations of GPs about the effectiveness of ABs discussed lead to varying views on the most appropriate treatment for their patients.

#### Affordability and availability of ABs within the healthcare system

When choosing a specific AB for TF treatment, not only subjective beliefs and previous experience of ABs effectiveness guided the choice, but also affordability and availability of ABs in the healthcare system. Although POMP is recommended as the first line of ABs in national recommendations for children, there is often limited availability of POMP.

“There would be a greater chance that we prescribed POMP if it was on the list of reimbursed medications...” (GP-6)

Currently, POMP is available for purchase in Latvia as an unauthorised medicine, which increases the price of this product and presents a risk of interrupted supply. Furthermore, POMP is not on the list of reimbursed drugs, and it is another reason why GPs who would have chosen to prescribe POMP do not prescribe it. In the case of TF, ABs are reimbursed only for children (up to 18 years of age). The list of reimbursed medications includes amoxicillin, amoxicillin/clavulanic acid, cefuroxime, cefprozil, and clarithromycin [33].

This factor of the healthcare system facilitates the prescription of broad-spectrum ABs when narrow-spectrum ABs would be sufficient. As a result, GPs follow rather pragmatic practice when subjecting their ABs choices to these healthcare system barriers for best practice standard.

### Choice of duration of AB therapy and dose

The choice of the duration and dose of AB therapy depended on (1) individual beliefs about the right therapy and (2) the size of the available ABs packaging within the healthcare system.

#### Beliefs about the right therapy

﻿Choice of the duration and dose of AB therapy depended on the beliefs of the GP about the right therapy. Some doctors thought that AB therapy should be short and in high doses. One of the GPs specifically indicated that the duration of POMP therapy should be 10 days, but in other cases the GPs did not choose a course of 10 days, because they have chosen to prescribe a broad spectrum ABs. A 10-day course for broad-spectrum penicillin, cephalosporin, or macrolides, as well as amoxicillin, was often implicated as unnecessary.

“I give short courses and large doses [about amoxicillin]. I never prescribe for 10 days to anyone, 7 days I give very, very rarely [about Amoxicillin]. On average, it would be up to 5 days, but for many, it is sufficient with 3 days.” (GP-7)

One of the GPs mentioned that he prescribes amoxicillin for 10 days in the case of a positive strep test. If the test result is negative, but the patient has symptoms, he prescribes a 5-day course of amoxicillin. This shows that doctors, even if the strep test is negative, tend to think that AB treatment is still necessary. Furthermore, it indicates that AB decision regarding the prescribing of AB is unstable, changing from one prescriber to another.

#### Available packaging sizes of ABs within healthcare system

The duration and dosage of therapy are also affected by a crucial factor of the healthcare system, the available packaging sizes of ABs. It is a very important factor that affects the length of POMP therapy because one package is enough for 4 days.

“[...] it is used 3 times a day for 10 days [about POMP], I know that sometimes I do wrong, because the packaging is for 4 days, and I prescribe for 8 days course. That packaging is not enough for 10 days.” (GP-1)

To complete a 10-day treatment course recommended by the guideline for TF, a patient should buy 3 full packages, but 2 packages would be sufficient for 8 days. The GPs thought that patients would not buy the third package and stop their treatment after 8 days. As a result, unsuitable packaging sizes of ABs, where an AB packaging cannot be divided, lead to a prioritized affordability over appropriate AB course length.

**Fig. 2:**
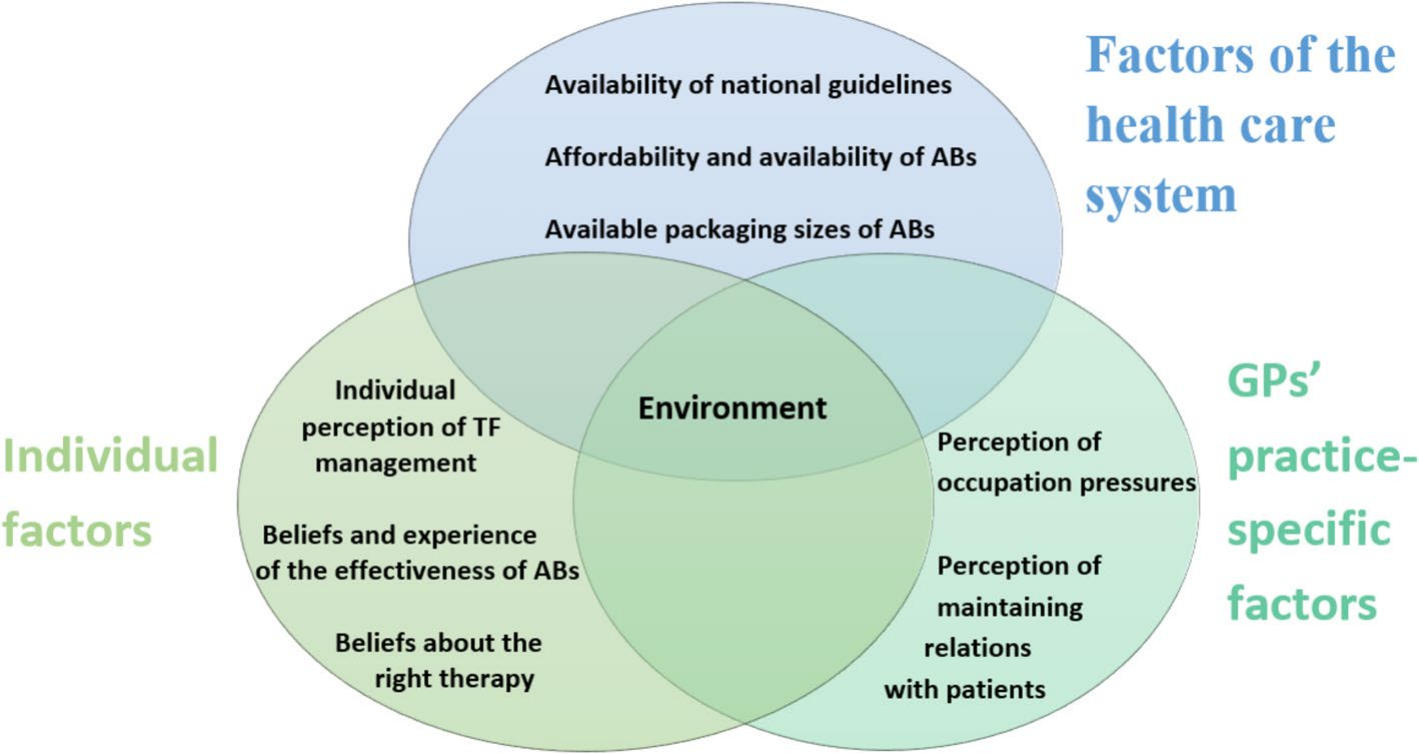
Analytical categories derived from thematic analysis

The concept developed from data after thematic analysis is available in Fig. 2. Diagnostic process of TF occurs within an environmental setting, where individual, practice-specific, healthcare system factors shape diagnosis and AB prescribing in TF.

### Strengths and limitations

A range of views was solicited, and data saturation was reached at the sixth of eighth interviews. Additionally, 2 interviews were conducted in which no new themes were identified. The findings offer insight into factors that influence the prescribing practice of GPs for TF in a primary care setting in conjunction with a specific context in which the prescriber practices. We had only one trained interviewer that can increase the reliability of the interviews.

The limitation of this study is that the interviewed GPs could systematically differ from those who declined to participate in this study. We did not have access to data that show the real prescribing rates for TF in GPs practices. There has been a long period of time between conducting the study and publishing the results, however, in Latvia reimbursement system has not changed, and guidelines are not available yet, which suggest that these results would still reflect the current practice in Latvia and serve as a case study to identify factors that influence the prescribing practice in a primary care setting in conjunction with a specific context in which the prescriber practices. We presume that overall results would be currently similar, as TF is diagnosis for which general practitioners prescribe AB most often. However, antibiotic consumption has substantially decreased between 2019 and 2020 in many EU/EEA countries, including Latvia. But it is not known whether the decrease is because of concomitant changes in disease transmission because of COVID-19 pandemic, healthcare utilisation patterns or prescription practices as there is a lack of patient-level data [34].

## Discussion

Our study showed that decisions and practice of the GPs are not static over time or context; they occur within an environmental setting influenced by individual factors of the GPs, the health care system, and the practice-specific factors that shape the diagnosis and prescribing factors in TF.

The most common factors of the health care system were the availability of ABs, the size of the AB package, and the availability of national guidelines.

Guidelines can improve the diagnostic process of GABHS TF and reduce unnecessary AB prescriptions. Adherence to the guidelines results in cost reductions, especially in terms of antimicrobial resistance (AMR) [5]. To reduce uncertainty in the diagnosis of TF it is reported that physicians in Sweden excessively use guidelines [28]. This contrasts with the practice observed in our study where practitioners work in an environment, where diagnostic resources such as official clinical guidelines or recommendations are not available.

Our study indicates that in the absence of national guidelines and consistency in health policy, GPs rely primarily on their personal experience and intuition (“gut feeling”) in making a diagnosis and choosing the most appropriate AB therapy. This was observed also in Saliba-Gustafsson et al. study, where lack of access to relevant AB prescribing guidelines was identified as a major barrier to appropriate AB prescribing [35]. From our study we cannot conclude that if there were a consistent health policy, evidence-based tools and resources in place that encourage rational AB prescribing, that would change the prescribing practice of GPs. The available literature shows that there are problems in following guidelines in clinical practice, also where they are available, such as in England and the USA. Some reports in the literature claimed that both NICE and US recommendations are at risk of being ignored in clinical practice. About 66% of physicians in a health care system fail to follow the guidelines [5, 36].

However, the available evidence also suggests that the environment plays an important role in enabling this behaviour. The study by Biezen et al. [37] showed that the use of guidelines is facilitated if there is a practice culture that encourages evidence-based practice.

Our study participants work in an environment where guidelines have marginal meaning, navigate diagnostic and treatment dilemmas within an environment with systematic health care system problems, which can encourage the misuse of ABs. This might be one of the reasons why behaviour changes in such an environment are harder to expect. Subsequently, GPs prescribe ABs as a habitual process within their clinical inertia, which could also be described as an adaptation to the environment. This environment can encourage practice, where evidence-based medicine is not at the center of the decision-making process, leading to the misuse of ABs.

Our study highlighted that the price and availability of ABs could influence the use of narrow-spectrum ABs. Prescription rates for penicillin V in Latvia compared to Scandinavian countries are very low [9, 38]. The availability and reimbursement status of medications (especially POMP) and the availability of easy-to-use guidelines can influence the practice of GPs. Health care system factors in this case facilitates a clinical practice that does not accept evidence-based medicine and the AMR concept and supports diagnostics as an individualised and inconsistent process for every patient.

The most common practice-specific factors identified in the study were occupation pressure and maintaining relationships with patients. This is in agreement with the results of previous studies [31], where Little et al. identified that social influences such as patient demand for ABs could influence AB prescribing. In cases where patients expect an AB prescription, the likelihood that a GP will provide it is higher. We observed similar patterns, where if patients’ want a shorter course of an AB, GPs followed patients’ preference. This is also consistent with Saliba-Gustafsson et al. results, where GPs’ perception of patients demands is a persistent issue that impacts prescribing decisions [35]. However, the study by Lum et al. that assessed the perspectives, attitudes, and behaviours of Australian consumers about ABs use indicated that most patients would accept the GP’s decision not to prescribe ABs if it was clearly explained [39]. In our study, GPs stressed that because of long working hours they are tired and consequently believed that AB prescribing might save time and make their workflow more efficient. In this case, an explanation of the rationale for prescribing or not prescribing ABs is missing, and the patient may not get all the information needed. This observation was also made in the Van der Zande et al. study [40], where physicians indicated that longer consultation time is needed when ABs are not prescribed and an additional support mechanism is needed. It indicates that in the system where crucial resources and support mechanisms are not implemented, it could be much harder to reduce unnecessary prescribing of ABs.

Furthermore, GPs feel pressure to shorten the duration of sick leave and to reduce patient TF symptoms faster. GPs expressed the need to ‘do something’, as parents want that their children or themselves return to school/kindergarten/work faster. Prescription of AB is an active action of the GPs, which not only aims to reduce sick leave, but also consultation time. In such practice, patients with bacterial TF would receive treatment faster, even if patients with viral TF would receive unnecessary AB treatment. This perception is misleading as published studies suggest that the reduction of sick leave cannot be achieved by AB prescribing, where Jiwa et al. concluded that the recovery of most patients who received ABs was similar to patients who did not receive treatment [41]. This finding further highlights important role of environment of rational AB prescribing.

The most common individual factors of the GPs were related to personal beliefs and previous experiences, such as the individual perception of TF management, beliefs and the experience of the ABs effectiveness and beliefs about the right therapy. They perceived the diagnosis of TF not to be complicated, but at the same time, the results showed uncertainty in the diagnosis process, which resulted in prescribing broad-spectrum ABs. GPs make a diagnosis of GABHS TF primarily empirically, although it is known that even the most experienced physicians might have difficulty clinically diagnosing GABHS pharyngitis [21, 42].

The interpretation of the most appropriate ABs and the duration of the necessary treatment in TF varied significantly between GPs. GPs relied primarily on their personal experience and the skills acquired in their practice, which were primarily driven by the environment. The study showed that the practice of GPs is encouraged by factors of the health care system, as well as their individual perception and experience. Our results are consistent with the findings of the Gröndal [16] study, showing that AB prescribing is an unstable category and rationalization of ABs is a complex process.

### Implications for future research, policy, and practice

For further research the next steps would be to investigate the impact of an environment, particularly the impact of health care system factors on AB prescribing practice in other indications, focusing on different environments in different countries. Deeper understanding of impact of environmental factors on AB prescribing in different countries would facilitate implementation of rational antibiotic use policy.

## Conclusion

The diagnosis of TF and the AB prescribing is inconsistent and unstable. Even if TF is considered by GPs a relatively easy diagnosis, the interviews showed that their decisions about prescribing differed and were contradictory. The environment in which GPs practice influence their prescribing practice, where afterwards decision regarding AB prescribing might become a clinical inertia, which is obtained behaviour.

TF is mostly treated empirically, and the introduction of easy-to-use clinical guidelines, in addition to increased awareness of the diagnosis and goals of AB therapy, could lead to more standardized diagnostic and prescribing practices.

## Data Availability

The data sets analysed during the current study are not publicly available but are available in Latvian from the corresponding author on reasonable request.

## Abbreviations

ABs: Antibiotics
AMR: Antimicrobial resistance
GPs: General Practitioners
GABHS: Group A beta-haemolytic streptococci
POMP: Phenoxymethylpenicillin
TF: Tonsillopharyngitis
RST: Rapid Strep Test
